# Anal Extrusion of Ventriculoperitoneal Shunt Distal Catheter: A Case Report and Literature Review

**DOI:** 10.2174/0115734056392417250628194723

**Published:** 2025-07-04

**Authors:** Jasmine Ahmed Alturaiki, Eissa Alousi, Mustafa Alhelal, Ali Alkhamees, Awn Alessa, Ibrahim Alahmed, Abdulsalam Mohammed Aleid

**Affiliations:** 1 Department of Surgery, King Faisal University, Al-Hofuf, Ahsa, Eastern Province, Saudi Arabia; 2 Department of Neurosurgery, King Fahad Hospital, Al-Hofuf, Ahsa, Eastern Province, Saudi Arabia; 3 Department of Neurosurgery, Medical College, King Faisal University, Al-Hofuf, Ahsa, Eastern Province, Saudi Arabia

**Keywords:** Ventriculoperitoneal shunt (VPS), Anal extrusion, Bowel perforation, Hydrocephalus, Myelomeningocele (MMC), Shunt migration, Pediatric neurosurgery, External ventricular drain (EVD), Rare complications, Catheter-related infections

## Abstract

**Background:**

The standard treatment for hydrocephalus is often the placement of a ventriculoperitoneal shunt (VPS), especially in patients with myelomeningocele (MMC). This case report aimed to enrich the existing knowledge by presenting a rare instance of asymptomatic anal extrusion of a VPS catheter in an infant, along with a review of the relevant literature.

**Case Presentation:**

A 2-month-old male infant with myelomeningocele (MMC) and hydrocephalus presented with asymptomatic anal extrusion of his ventriculoperitoneal shunt (VPS) catheter, discovered by his mother. Emergency imaging revealed distal catheter migration through the rectosigmoid junction. Surgical management included (1) laparoscopic-assisted catheter removal with bowel repair using Vicryl sutures, (2) intraoperative external ventricular drain (EVD) placement, and (3) 14-day antibiotic prophylaxis. Cerebrospinal fluid analysis remained normal throughout the treatment. Following three weeks of infection monitoring, contralateral VPS replacement was performed successfully, with postoperative imaging confirming optimal shunt function and resolved hydrocephalus. This case highlighted the importance of caregiver vigilance in identifying this rare but serious complication, even in asymptomatic patients (Fig. **1**).

**Conclusion:**

Although anal extrusion of a VPS catheter is an uncommon but serious complication, primarily seen in pediatric patients, it can lead to life-threatening infections if untreated. Prompt surgical intervention along with broad-spectrum antibiotic therapy is critical. This report highlights the need for recognizing classic symptoms of intestinal perforation and catheter migration in pediatric patients.

## 1 INTRODUCTION

Hydrocephalus is marked by elevated intracranial pressure due to an abnormality in cerebrospinal fluid (CSF) flow. It is categorized into two types: communicating and non-communicating hydrocephalus. Treatment options include CSF shunting, endoscopic third ventriculostomy (ETV), and choroid plexus cauterization; however, the ventriculoperitoneal shunt (VPS) is the most widely used surgical intervention. Although VPS is generally effective, it is associated with a range of complications, including shunt malfunction, blockage, infection, and over-drainage, as well as less frequent issues, such as spontaneous bowel perforation or shunt migration, which can lead to catheter extrusion through the anus, mouth, or vagina. In rare cases, catheters may migrate to the heart. This case report highlights the case of an infant with asymptomatic anal extrusion of a VPS catheter.

## 2 CASE REPORT

A mother noticed the VPS catheter protruding from the anus of her 2-month-old son, a male infant with a history of MMC and hydrocephalus. He was immediately taken to the emergency room. Despite the extrusion, the patient remained asymptomatic and exhibited no signs of systemic illness or gastrointestinal complications. Imaging confirmed that the distal catheter had penetrated the rectosigmoid junction. The distal VPS catheter was surgically removed *via* laparotomy. During the procedure, the catheter was carefully severed at the mid-abdomen to minimize further trauma to the bowel. The protruding anal segment was gently extracted through the anus without resistance after confirming the extent of bowel perforation. The affected bowel segment was repaired using medium-sized Vicryl sutures. An external ventricular drain (EVD) was inserted intraoperatively to manage intracranial pressure. Cerebrospinal fluid (CSF) obtained from the EVD showed no evidence of infection; cultures were sterile, and routine analysis revealed normal glucose, protein, and cell counts. The patient was treated with intravenous broad-spectrum antibiotics for 14 days, which were discontinued following clinical improvement and negative repeat CSF cultures. After three weeks of monitoring and confirming the absence of infection, a new ventriculoperitoneal shunt was successfully placed on the contralateral side. Postoperative neuroimaging confirmed appropriate shunt placement, satisfactory ventricular decompression, and resolution of hydrocephalus.

### 2.1 Medical, Family, and Psychosocial History

The newborn, diagnosed with myelomeningocele (MMC), required urgent surgery to address hydrocephalus. The mother had poor dietary habits during pregnancy, including insufficient folic acid consumption, though no intrauterine infections were reported. After birth, the infant underwent both VPS placement and surgical repair of the MMC. There was no family history of consanguinity, hydrocephalus, or neural tube defects. When faced with these challenges, the caregivers responded promptly, ensuring a stable psychosocial environment.

**Fig. (1):**
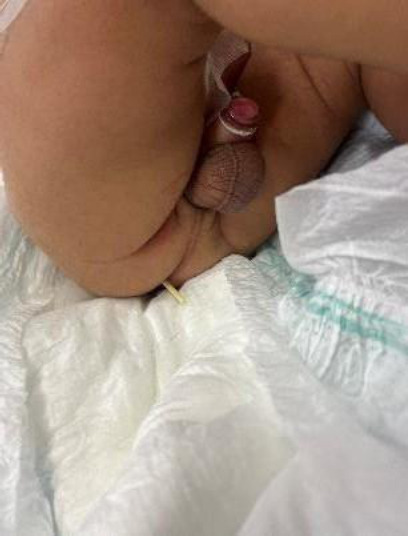
Distal shunt catheter extrusion through the anus.

The infant exhibited normal feeding and activity, with no fever or gastrointestinal issues. On physical examination, the anterior fontanelle was soft, head circumference was normal, vital signs were stable, and no meningeal irritation was present. The abdomen was distended and tympanic on percussion. Approximately 5 cm of the distal catheter protruded from the anus, with CSF leaking from the catheter (Fig. **1**).

### 2.2 Timeline of the Case

Day 0 (birth): The patient underwent surgical repair of MMC within 72 hours of birth, followed by the immediate placement of a medium-pressure VPS.

Day 60 (2 months later): The patient was brought to the emergency department after the mother noticed a portion of the VPS catheter protruding from the anus.

**Fig. (2):**
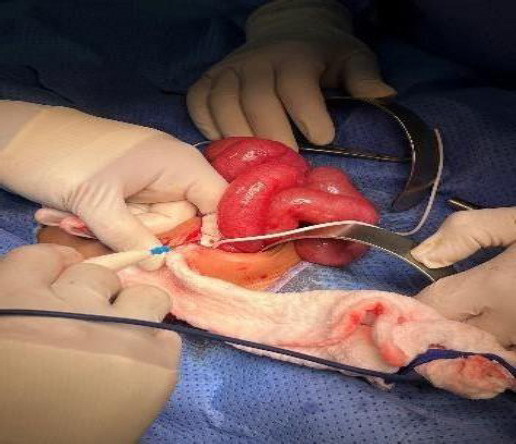
Bowel distension and obstruction caused by the looped catheter.

Day 61: Physical examination and imaging revealed signs of bowel obstruction and a perforation at the rectosigmoid junction, involving the distal catheter. Emergency surgery was performed to manage this complication (Fig. **2**).

**Fig. (3):**
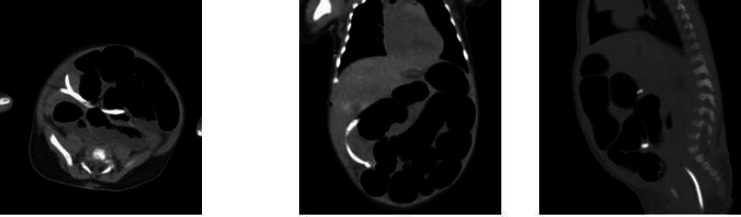
Axial, coronal, and sagittal CT views showing that the distal catheter had looped round the ilium resulting in dilated bowel and perforated rectum and migrated through anal verge.

A computed tomography (CT) scan of the brain showed no evidence of acute hydrocephalus. However, the abdominal CT revealed that the distal portion of the catheter had perforated the rectosigmoid junction and extended through the anal verge. Additionally, multiple small bowel loops demonstrated air-fluid levels, and bowel dilation was observed (Fig. **3**).

An urgent laparotomy-assisted procedure was performed to remove the VPS and place an external ventricular drain (EVD). CSF samples were collected intraoperatively from both the proximal valve and the distal intra-abdominal catheter. Microbiological cultures yielded no bacterial growth, and CSF analysis showed normal cell count and glucose and protein levels, effectively ruling out ventriculitis. The extruded distal catheter, which had protruded through the anus, was carefully removed surgically under general anesthesia through an abdominal incision. The peritoneal portion was disconnected and extracted in a controlled, sterile environment to minimize the risk of peritoneal contamination.

Following the removal of the infected hardware, an external ventricular drain (EVD) was inserted, and CSF was monitored. Given the absence of infection, empirical intravenous antibiotics (vancomycin and ceftriaxone) were administered for 7 days, after which repeated CSF cultures remained sterile, and inflammatory markers were normalized. A new ventriculoperitoneal (VP) shunt was subsequently placed on the contralateral side, ensuring no reuse of previous shunt tracks. The patient had no postoperative complications and was discharged in stable condition.

**Fig. (4):**
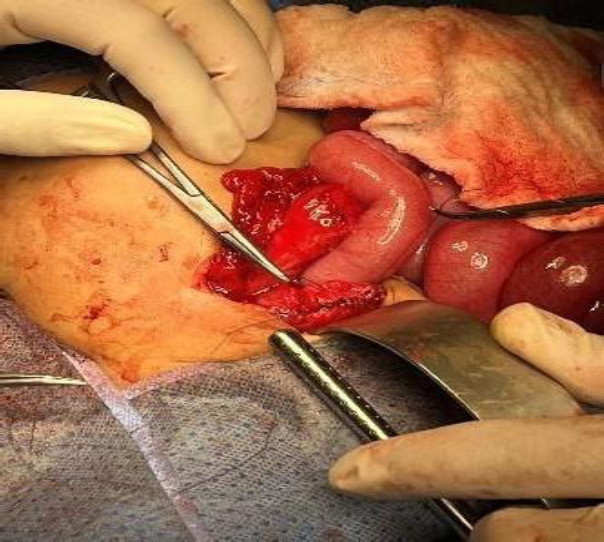
Primary repair of perforated rectosigmoid junction.

The neurosurgery and pediatric surgery teams worked together to complete the procedure. The proximal catheter, valve, and cranial entry point were excised, and the EVD was inserted. The peritoneal catheter had coiled around the ileum, contributing to bowel dilation, and had penetrated the rectosigmoid area, ultimately passing into the rectum (Fig. **4**). The distal catheter was severed at the mid-abdomen, releasing the bowel, and the perforated bowel segment was repaired using medium-size Vicryl sutures.

### 2.3 Postoperative Timeline

Days 62–76: After surgery, the patient received intravenous (IV) broad-spectrum antibiotics for 2 weeks. Antibiotic therapy was discontinued after 14 days of IV treatment upon resolution of systemic inflammation (CRP <0.5 mg/dL, afebrile ×72 hours) and confirmation of sterile follow-up CSF cultures. The external ventricular drain (EVD) was removed on hospital day 10 after clamping trials demonstrated stable intracranial pressure (<15 cm H_2_O). Subsequent CSF analysis revealed WBC 8/mm^3^ (lymphocyte-predominant), glucose 52 mg/dL (serum: 98 mg/dL), protein 38 mg/dL, and negative Gram stain/cultures.

**Fig. (5):**
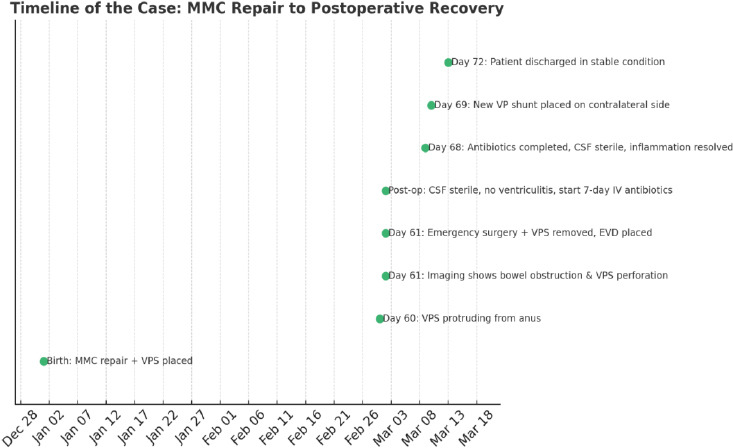
Timeline of the case: MMC to postoperative recovery.

The protruding anal catheter was surgically excised on hospital day 7 under general anesthesia. The distal intra-abdominal catheter segment exhibited purulent material (*C. acnes* on culture); the proximal ventricular catheter tip cultures were sterile (Fig. **5**). The surgical site was irrigated with vancomycin (5 mg/mL), and a new ventricular catheter was tunneled to a clean abdominal quadrant.

A permanent ventriculoperitoneal (VP) shunt (medium-pressure valve) was placed on hospital day 17, utilizing intraoperative ultrasound to confirm optimal placement. This timeline adhered to institutional protocol requiring (1) 7 days of negative CSF cultures post-EVD removal, (2) normalization of CSF profiles, and (3) absence of abdominal abscess on CT. The patient exhibited no signs of recurrent infection at 30-day follow-up.

The patient was maintained nil per os (NPO) for 72 hours with intravenous (IV) fluid support. Serial abdominal examinations demonstrated progressive resolution of distension.

**Fig. (6):**
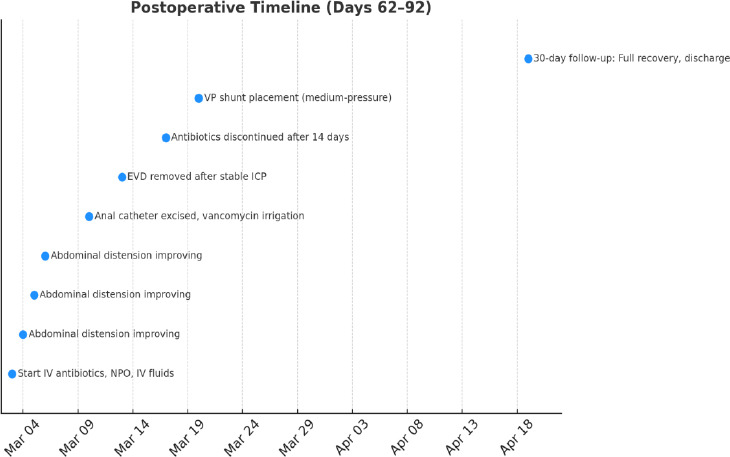
Timeline of the case: postoperative timeline (days 62-92).

Day 76: During the follow-up visit, the patient fully recovered and was discharged without any complications (Fig. **6**).

### 2.4 Diagnostic Challenges

The diagnosis of ventriculoperitoneal shunt (VPS) catheter migration with concurrent intestinal perforation presented considerable diagnostic challenges. In this case, the diagnosis was relatively straightforward as the catheter was visibly protruding from the patient’s anus. However, early signs of infection, such as fever or abdominal pain, were absent. Notably, only about 25% of patients with bowel perforation manifest clinical signs of peritonitis, with asymptomatic presentations being particularly common in ventriculoperitoneal shunt (VPS) cases. Imaging studies, particularly a CT scan, are necessary to confirm the catheter’s location and the extent of the intestinal perforation. There is also a risk of meningitis or ventriculitis developing without prior symptoms, making early diagnosis crucial.

### 2.5 Prognosis

When diagnosed and treated early, intestinal perforation and VPS catheter migration have a generally favorable prognosis. In the present case, no postoperative complications were observed following prompt surgical management, including bowel repair, EVD placement, and catheter removal. However, delayed treatment can lead to more severe outcomes, with a 15% risk of meningitis or ventriculitis. Long-term follow-up indicated a favorable prognosis for this patient, with no signs of infection or neurological damage. Nevertheless, continued monitoring is essential, especially for patients with underlying conditions, like MMC, which may predispose them to future complications.

### 2.6 Patient Perspective

When the patient’s family observed the catheter protruding from the anus, they were understandably alarmed. However, they expressed great satisfaction with the prompt and attentive care provided by the medical team. Continuous multidisciplinary team communication and psychosocial support throughout both the acute emergency and convalescent phases significantly alleviated caregiver distress. The family followed all post-surgical instructions diligently, including adherence to the NPO protocol and antibiotic regimen. The parents expressed profound relief as their child achieved complete recovery with no complications observed during longitudinal follow-up. They also greatly appreciated the collaboration between the pediatric surgery and neurosurgery teams, which gave them confidence in the care their child received.

## 3 DISCUSSION

MMC, often referred to as open spina bifida, is a congenital abnormality of the central nervous system in which the spinal cord and meninges protrude through defective vertebral arches [1]. MMC is closely linked to inadequate folic acid intake during pregnancy [2], with hydrocephalus present in approximately 85% of cases [3]. Standard treatment involves surgical closure of the lesion and placement of a ventriculoperitoneal shunt (VPS) within the first 48 hours after birth [4].

While ventriculoperitoneal shunting (VPS) remains the gold-standard treatment for hydrocephalus, the procedure carries significant inherent risks, including infection, malfunction, and rare but serious complications, such as intestinal perforation. The most frequent complications are shunt obstruction and infection, which typically occur within the first few months following insertion [5]. Although uncommon, bowel perforation represents a recognized complication of ventriculoperitoneal shunting, with reported incidence rates ranging from 0.1% to 0.7% in published series [4]. Shunt migration is another uncommon issue, where the catheter moves to the anus, mouth, or vagina [6]. There are 139 documented cases of anal extrusion in the literature.

In the present case, ventriculoperitoneal shunt (VPS) catheter migration occurred within 2 months post-implantation, consistent with the documented migration window of 2-20 months [7]. Notably, this complication demonstrates a male predominance, with males comprising 68% of reported cases [8].

While the precise pathophysiology of catheter migration remains incompletely understood, multiple predisposing factors have been proposed, including (1) structural weakness of the bowel wall, (2) mechanical factors (*e.g*., sharp catheter tips or inappropriate catheter length), (3) chronic inflammatory processes, (4) surgical history, (5) infectious complications, and (6) silicone hypersensitivity reactions [9].

In this case, the VPS catheter migrated within 2 months of placement, aligning with the reported migration window of 2-20 months [10]. Notably, this complication disproportionately affects males, who account for approximately 68% of reported cases [11]. While the exact pathophysiological mechanism remains unclear, several factors are believed to contribute, including weak bowel musculature, sharp catheter edges, excessive catheter length, chronic irritation, history of abdominal surgeries, infections, and silicone allergy [12].

A widely cited explanation for gastrointestinal perforation caused by VPS was proposed earlier [13]. The theory emphasized the role of chronic aseptic inflammation and mechanical stress. According to the model, the catheter, once introduced into the peritoneal cavity, induces local inflammatory responses that lead to fibrous tissue formation and fixation of the catheter. Persistent mechanical irritation at the same bowel segment then weakens the bowel wall over time, eventually leading to perforation.

The present case added to Rubin et al.’s hypothesis. During surgery, fibrous adhesions and inflammatory tissue were observed surrounding the distal catheter, consistent with chronic irritation and localized inflammation. This fibrotic encapsulation likely fixed the catheter in position, enhancing the mechanical pressure exerted on a single point of the anterior bowel wall. Additionally, the patient’s underlying MMC may have contributed to altered intra-abdominal dynamics or congenital weakness in the bowel wall, further facilitating catheter erosion and eventual migration.

Our case, therefore, not only reaffirmed Rubin et al.'s mechanism of perforation, but also highlighted how anatomical abnormalities associated with MMC may create a predisposed environment for such rare complications. Awareness of these underlying vulnerabilities is crucial for early recognition and timely surgical intervention in similar cases.

### 3.1 Clinician/Patient-assessed Outcomes

The patient demonstrated an excellent postoperative recovery, with complete resolution of abdominal distension and no neurological sequelae or surgical complications. Throughout the follow-up period, close monitoring revealed no clinical evidence of meningitis, peritonitis, or other shunt-related complications, consistent with prior reports [14]. The patient successfully tolerated diet advancement to regular oral intake, with caregivers reporting no concerns during subsequent outpatient evaluations. During follow-up visits, no complications were observed, and the patient was discharged with an overall improved condition.

### 3.2 Follow-up Tests

In the absence of clinical red flags, follow-up imaging was deemed unnecessary. Regular postoperative monitoring, consisting of detailed neurological assessments (including cranial nerve evaluation and motor function testing) and abdominal examinations, confirmed complete symptom resolution and absence of shunt-related complications. Previous studies have emphasized the importance of imaging tests, such as CT scans or ultrasounds, in cases where hydrocephalus symptoms warrant further investigation [15].

### 3.3 Intervention Adherence/Tolerability

Postoperative compliance was exemplary, with strict adherence to the prescribed 72-hour nil per os (NPO) protocol and completion of the full 14-day course of intravenous broad-spectrum antibiotic therapy. The patient tolerated both external ventricular drain (EVD) placement and antimicrobial treatment without complications. Caregivers demonstrated meticulous compliance with all medical recommendations, facilitating an uneventful transition from parenteral to enteral nutrition. Owing to the absence of observed neurological or gastrointestinal side effects, the therapies were considered safe and effective.

Management of VPS catheter extrusion through the anus typically involves catheter removal, insertion of EVD, and antibiotic treatment. In cases where bowel perforation is present, an exploratory laparotomy and bowel repair are required, as in the present case.

### 3.4 Recommendations

This case underscored the critical importance of maintaining a high index of suspicion for ventriculoperitoneal shunt (VPS)-related intestinal perforation and catheter migration, even in asymptomatic patients. Early radiographic confirmation of catheter position and thorough evaluation for occult bowel injury are essential, as these complications frequently present with subtle or absent clinical findings. Managing such complex cases poses a significant challenge to surgeons, necessitating multidisciplinary collaboration with other specialists, including neurosurgeons and pediatric surgeons. Patients with myelomeningocele (MMC) necessitate comprehensive longitudinal follow-up and scrupulous postoperative management due to their substantially elevated risk of complications. Effective moisture control during the initial postoperative period represents a critical intervention for enhancing long-term prognosis and reducing the incidence of severe CNS infections, including meningitis and ventriculitis.

## CONCLUSION

Anal extrusion of a ventriculoperitoneal shunt (VPS) catheter with associated intestinal perforation constitutes an uncommon yet potentially life-threatening complication that predominantly occurs in the infant population, frequently presenting with minimal or absent clinical symptoms. The standard therapeutic approach encompasses (1) immediate shunt extraction, (2) placement of an external ventricular drain (EVD), and (3) administration of broad-spectrum intravenous antibiotics. In cases complicated by peritonitis or other severe manifestations, exploratory laparotomy with definitive bowel repair is imperative. Early recognition and expeditious surgical management are essential to mitigate the risk of devastating neurological sequelae, including meningitis and ventriculitis.

## STUDY LIMITATIONS

This report has described a single patient experience, limiting generalizability. Imaging findings were limited, and long-term follow-up data were not available. Further case accumulation is needed to refine management approaches.

## AUTHORS’ CONTRIBUTIONS

The authors confirm their contribution to the paper as follows: J.A.: Study conception and design; EA, MA, AAK, AAE, IA, AA: Writing, reviewing, and editing. All authors have reviewed the results and approved the final version of the manuscript.

## Data Availability

All the data and supporting information are provided within the article.

## LIST OF ABBREVIATIONS

VPS: Ventriculoperitoneal shunt
MMC: Myelomeningocele
CSF: Cerebrospinal fluid
EVD: External Ventricular Drain
NPO: Nil Per Os (nothing by mouth)
CT: Computed Tomography
IV: Intravenous

## STANDARDS OF REPORTING

CARE guidelines were followed.
